# Molecular profiling of breast cancer: clinical implications

**DOI:** 10.1038/sj.bjc.6601667

**Published:** 2004-03-02

**Authors:** S Cleator, A Ashworth

**Affiliations:** 1 The Breakthrough Breast Cancer Research Centre, Institute of Cancer Research, Fulham Road, London SW3 6JB, UK

**Keywords:** breast cancer, microarray, profiling, prognosis

## Abstract

Breast cancers are routinely subcategorised on the basis of clinical stage, cellular morphology and immunohistochemical analysis of a small number of markers. The recent development of gene expression microarray and related technologies provides an opportunity to perform more detailed profiling of the disease. It is anticipated that the molecular classification arising from such studies will be more powerful than its pathological predecessor at confining treatment to those patients who are most likely to benefit. It is likely that this will result in a much less frequent use of adjuvant chemotherapy. Furthermore, of those who do receive it, a higher proportion will benefit. If adopted, this will offer considerable patient benefits in terms of reducing unnecessary toxicity and have major health economic implications.

Most cancer types, and breast cancer is no exception, can be subcategorised by clinical stage and pathological subtype. These categories can be correlated with survival data, which allows the prediction of disease natural history and, to a lesser extent, treatment response and benefit for a given patient. This is fundamental to therapeutic decision-making in oncology and increasingly allows treatment to be tailored on an individual patient basis.

However, the standard methods for subtyping breast cancers remain relatively crude. Clinical staging and routine pathology are the principle indices used to identify those individuals at risk of developing metastases and who should therefore be considered for adjuvant chemotherapy. These features have been incorporated into various standardised scoring algorithms (Galea *et al*, 1992; Goldhirsch *et al*, 1998; Eifel *et al*, 2001). Their application, however, results in the ‘over-treatment’ of many patients in whom cure would have been achieved without chemotherapy or possibly even endocrine treatment. This is illustrated by the Oxford Overviews of systemic treatments that demonstrate a significant proportion of long-term survivors in the untreated arms (Early Breast Cancer Trialists' Collaborative group, 1998a).

Another group of patients who receive treatment from which they will not benefit are those who will develop metastatic disease despite adjuvant cytotoxic treatment. Although staining for the oestrogen and HER-2 receptors are powerful individual predictors of response (and benefit from) tamoxifen and herceptin, respectively, clinicians lack a marker that predicts those who will benefit from chemotherapy.

Unlike standard methodologies that rely on a few pathological features and immunohistochemical markers, molecular profiling allows tumours to be defined by the expression pattern or genomic alteration of thousands of genes simultaneously. With these techniques comes the prospect of defining individual genes or combinations of genes whose expression level(s) can discriminate efficiently between clinically significant subtypes of breast tumours requiring different treatment strategies.

Gene expression microarrays have been used extensively to study breast cancer. The technical aspects of these approaches have been reviewed extensively in the scientific literature (Schulze and Downward, 2001). Here, we will consider the role of expression microarray profiling in the definition of existing and novel categories of breast cancer. In particular, we will address how it may reduce the large number of breast cancer patients who receive inappropriate, yet toxic treatments.

## GENE EXPRESSION MICROARRAY EXPRESSION PROFILING TO DEFINE SUBCATEGORIES OF BREAST CANCERS

Broadly speaking, subclassification of cancers by gene expression microarray analysis can be performed in one of two ways. Microarray data from a selection of clinical samples may be interrogated for ‘clusters’ of samples that are statistically significantly related in terms of their expression profiles. Samples that share expression profile features might be expected to share phenotypic features such as those that can be clearly defined pathologically, for example oestrogen receptor (ER) status, or those that are less obvious, for example chemosensitivity. This approach is referred to as an *unsupervised analysis* (or clustering) (Quackenbush, 2001). In contrast, a *supervised analysis* begins with designation of the samples to ‘labelled’ phenotypic subcategories. A search is then made to define a list of genes that are distinct in their expression between the two ‘labelled’ groups and those that can be used to distinguish between them. The discriminatory accuracy of the list of genes defined in this way can then be tested for its ability to separate the samples into the defined groups on an independent set of samples (a ‘validation set’). For example, the expression profile of samples from chemosensitive and chemoresistant breast tumours can be compared. A list of genes that are differentially expressed between the two groups is obtained and then assessed for its ability to predict response in a separate group of samples (Quackenbush, 2001).

Initial studies set out to demonstrate that breast cancers with distinct pathological features could be ‘separated’ by microarray. Several groups demonstrated that supervised data analysis can be used to derive a set of genes that can distinguish ER-positive cancers from ER-negative tumours (Gruvberger *et al*, 2001; West *et al*, 2001; van ‘t Veer *et al*, 2002). While these studies were important in validating the technology and developing the methodology for data analysis, extensive expression profiling of this nature is unlikely to replace standard immunohistochemical assessment of ER status; smaller customized arrays, however, consisting of tens not thousands of genes, or customized real-time quantitative PCR platforms (‘gene-cards’) represent a more realistic prospect as a clinically useful assay. These studies also demonstrated, perhaps initially somewhat surprisingly, just how different ER-positive breast cancer is from ER-negative cancers. The fact that ER-positive and ER-negative tumours are so different at the level of gene expression, suggests that these molecular subtypes are entirely different disease entities, which perhaps arise from distinct precursor cell types. Furthermore, only a few of the genes that discriminate between ER-positive and ER-negative tumours appear to be part of the ER signaling pathway, adding further weight to the concept of distinct lineages for ER-positive and ER-negative tumours (Gruvberger *et al*, 2001).

Known pathological subtypes can also be identified by clustering using a selected panel of genes. In a recent study (Sorlie *et al*, 2003), which is an extension of the earlier work (Perou *et al*, 2000; Sorlie *et al*, 2001), 115 malignant breast tumours were analysed by hierarchical clustering and were shown to subdivide into five subgroups, some of which had been previously recognised and some of which were new entities. The distinction was greatest between tumours showing high expression of ‘luminal epithelial specific genes’, including ER, and those not expressing these genes.

## MOLECULAR PROFILING TO PREDICT DISEASE RELAPSE

The use of gene expression microarray analysis has an obvious potential role as a means of predicting relapse. The molecularly defined sub-types of breast cancer mentioned above (Sorlie *et al*, 2003) were associated with differences in clinical outcome. This suggests that subtypes derived by an unsupervised analysis are indeed of clinical significance. Nevertheless, a list of discriminatory genes useful as a ‘test’ of prognosis, is best obtained by a supervised analysis. In a landmark study, van ‘t Veer *et al* (2002) performed gene expression microarray analysis on 78 sporadic lymph-node-negative tumours arising in women under the age of 55 years. Approximately half of the tumours had given rise to metastases at 5 years. The study was particularly informative as very few of the patient groups had received systemic treatment, which is likely to benefit a molecular subset of patients and thereby modify the outcome. Microarray analysis of these primary tumours identified a list of 70 discriminatory genes whose expression patterns, in an internal validation, identified a group of patients who had not developed metastases at 5 years from diagnosis despite no systemic treatment.

**Figure 1:**
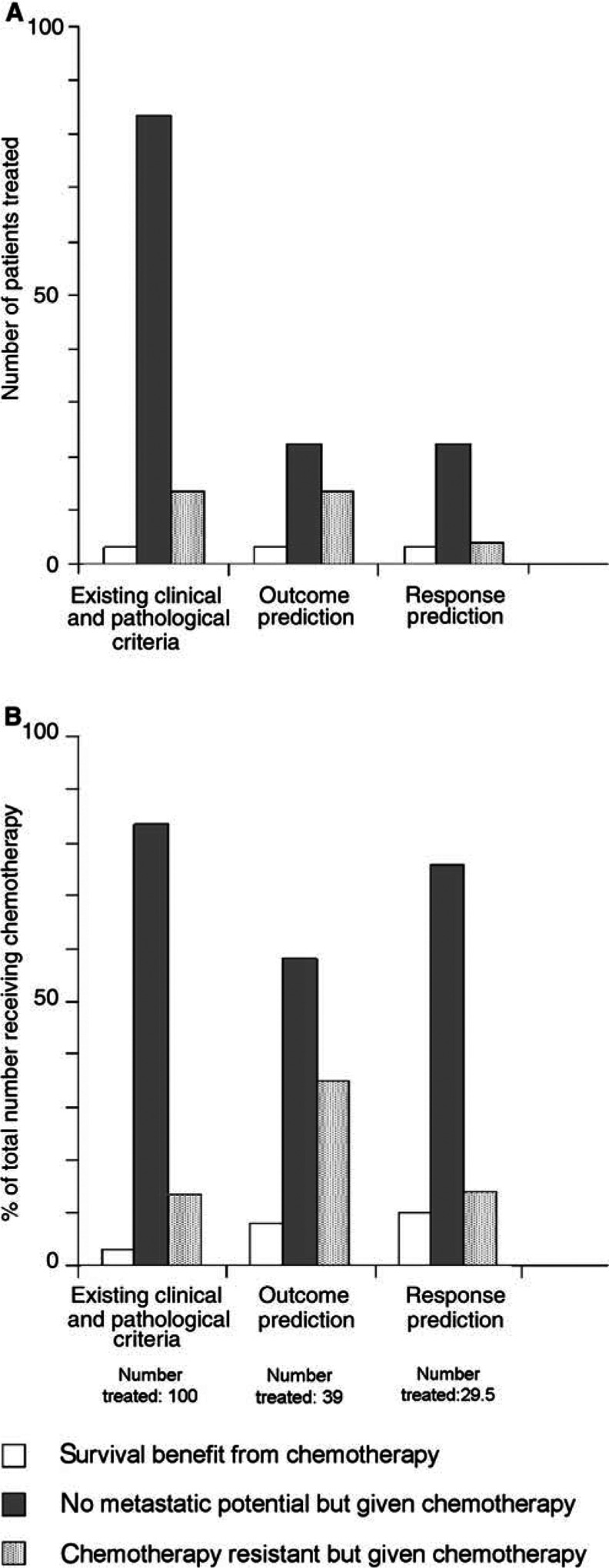
Model for the effect of molecular profiling on numbers of premenopausal women with node negative breast cancer receiving chemotherapy, and associated benefit at 5 years. (**A**) The Oxford overview of polychemotherapy (Early Breast Cancer Trialists' Collaborative group, 1998b) indicated that for 100 node-negative, premenopausal women receiving chemotherapy according to standard criteria, at 5 years three are cured by chemotherapy, 83.5 would have been alive without chemotherapy and 13.5 die despite chemotherapy. With application of molecular profiling to predict the outcome, these figures (for the same 100 people) would become 3, 22.5 (false-positive rate of 27% from van ‘t Veer *et al*, 2002) and 13.5, respectively. If response could be predicted, the third column would be reduced further to the false-positive rate for the predictive profile used (here we have assumed that this is approximately as for outcome profiling, that is 27%). The second column may be reduced but this is difficult to estimate. This illustrates that much less chemotherapy would be given. (**B**) This shows the proportional benefit of women receiving chemotherapy according to the same criteria. Considering the same 100 women, if outcome prediction with molecular profiling of breast cancer was employed, the number treated would be reduced to 39, resulting in an increase in the proportion cured (from three out of 100 to three out of 39 or 8%). If it were possible to predict chemoresponsiveness, it is possible that the number receiving chemotherapy would reduce further from 39 to 29.5 (allowing for a false-positive rate equivalent to that seen in the van ‘t Veer study). A reduction in the second column would probably occur but is not shown. In this scenario, the proportion cured by chemotherapy would be three out of 29.5, approximately 10% (a three-fold increase), and the number of women treated has been reduced by 70%. Note that in neither figure has consideration been given to the false-negative rate inherent in molecular profiling. It has been assumed that all deaths occurring were breast cancer related.

A method such as this for identifying patients at no risk of developing metastases has the potential to reduce markedly the overall amount of chemotherapy prescribed and to increase the proportion of women who benefit from it (see Figure 1

). At present, the majority of premenopausal women with node-negative breast cancer receive adjuvant chemotherapy, but the absolute survival benefit from treatment (proportion cured by the use of chemotherapy) is only around 3% at 5 years (Early Breast Cancer Trialists' Collaborative group, 1998b). In other words, 33 women are treated to save one life. (The benefit is greater at 10 years, 5.7%, but we have used the 5-year figures to correspond with the van ‘t Veer study.) In the corresponding control arm, survival was 83.5% at 5 years without chemotherapy. If these women were correctly identified by molecular profiling with 100% accuracy, and effectively removed from the treatment arm, the survival benefit would be much greater; three lives for 16.5 treated (one life/5.5 treated) (see Figure 1). The van ‘t Veer analysis was associated with a false-positive rate of 27% at 5 years (‘nonmetastatic’ patients incorrectly assigned to the high-risk ‘metastatic group’). Therefore, with this level of misassignment, the survival benefit would be three lives per 39 treated (one life/13 treated).

However, consideration should be given to the fact that there was a false-negative rate of 9% in the poor prognosis group, that is patients incorrectly assigned to the ‘no metastases’ group, which would have resulted in their ‘undertreatment’. Furthermore, clinical follow-up on this study stands at 5 years, while breast cancer has the potential to recur beyond this time point. It will be interesting to observe the clinical course of the surviving patients over the next 5 years in the context of their expression profile.

Positive pathological node status is strongly associated with poor outcome in breast cancer, but is by no means a guarantee of systemic relapse. Other groups have used a similar method to predict pathological lymph-node status from the expression profile of the primary tumour (West *et al*, 2001; Huang *et al*, 2003). While this is of interest, this is unlikely to be adopted as a clinical tool because lymph-node status is unsatisfactory as a surrogate of the outcome and what is required is a test of superior predictive power.

Concerns regarding the risk of ‘under treatment’ of women with metastatic potential, short clinical follow-up, a lack of prospective clinical validation and limitations regarding access to this technology means that the use of this approach is some way off being adopted as a routine test. However trials are planned to assess prospectively the validity of this technique in assigning adjuvant treatment, and it is certain that it does represent a genuine prospect for the future. Importantly, the new trials will not only generate the statistically significant patient numbers to validate microarray-derived prognostic signatures but also have the potential of providing data that will allow for the refinement of such signatures.

## PREDICTING CHEMOTHERAPY RESPONSE

There is evidence to suggest that response to neoadjuvant (presurgical) chemotherapy can be used as a surrogate of chemotherapy survival benefit (Smith and Lipton, 2001). Several studies have shown that complete pathological response predicts for improved overall survival and good clinical response predicts for improved disease-free survival (Cleator *et al*, 2002). It is also reasonable to assume that good response in the neoadjuvant setting indicates survival benefit in the adjuvant setting. There are many descriptions of potential predictive markers of response to neoadjuvant chemotherapy. However, as yet none of the markers studied have proved of sufficient discriminatory power to be employed in a clinical setting. It is anticipated that the supervised analysis of gene expression microarray data from responsive compared to nonresponsive tumours may allow the definition of a panel of clinically useful discriminatory genes.

Preliminary data published in this area are promising but are hampered, as is frequently the case, by small sample size (Sotiriou *et al*, 2002; Chang *et al*, 2003; Pusztai *et al*, 2003). In the largest of these studies, Chang *et al* performed expression arrays on samples from 24 patients, 11 of which were classified as chemosensitive and 13 of which were classified as chemoresistant. Of note these two groups were not balanced in terms of tumour size or pathological type, both of which may influence tumour response. A total of 92 genes selected as a discriminatory gene set predicted response correctly in six out of six independent samples (all responders). The inherent statistical error arising from the analysis of thousands of gene expression ratio readings on such a small (comparatively) number of samples can be considerable, so these results should be validated on a larger data set. It is important to note that this set of genes may apply only to sensitivity to a particular agent (docetaxel) or class of agent (taxanes), and it seems possible that specific gene sets will need to be developed to predict sensitivity to individual cytotoxic treatments. However, the use of different microarray platforms in deriving the existing data sets makes this difficult to assess at this time (Lee *et al*, 2003).

If accurate determination of chemosensitivity were achieved by this means, the overall number receiving cytotoxic treatment unnecessarily would decrease, and the overall survival benefit derived, per person treated, increase accordingly, as shown in Figure 1. However, the absolute survival benefit of patients diagnosed with breast cancer would be unaffected and will only improve if more effective agents are developed.

## MOLECULAR PROFILING TO PREDICT PROGRESSION OF DUCTAL CARCINOMA *IN SITU*

Several studies have shown that the molecular profile of the primary tumour can predict the future development of metastases (van ‘t Veer *et al*, 2002; Huang *et al*, 2003). This suggests that propensity to metastasize is a feature determined early in tumorigenesis (Bernards and Weinberg, 2002). In a similar way, it is possible that ductal carcinoma *in situ* (DCIS) develops the features that result in progression to invasive disease early in the pathway of tumorigenesis. Indeed, microdissection of breast cancers to isolate atypical ductal hyperplasia (ADH), *in situ* and invasive components reveal very similar molecular profiles at all stages, both in terms of expression (Ma *et al*, 2003) and chromosomal aberrations (Aubele *et al*, 2000). Administration of adjuvant radiotherapy following local excision of focal DCIS results in a reduction in the incidence of development of invasive relapse (Houghton *et al*, 2003). However, it is clear that even without radiation many patients do not relapse. This may be either because all DCIS present were excised or because any DCIS remaining failed to progress (and these patients did not develop new lesions). It is likely that only certain subtypes of DCIS will progress to invasive disease and therefore derive benefit from radiotherapy. Theoretically it should be possible to identify a DCIS expression profile that predicts a high probability of progression to the invasive form of the disease, which could be used to target adjuvant treatment more accurately than is achieved using clinical and pathological scoring systems alone (Douglas-Jones *et al*, 1996). However, these studies will be difficult to perform as many patients with the so-called focal DCIS will in fact have multiple, genetically diverse undetected lesions that follow a different natural history.

## CONCLUSIONS AND NEW HORIZONS

At present, many women with breast cancer undergo lengthy treatments with acute and long-term toxicity, but no benefit in survival. More accurate prediction of prognosis would significantly reduce the amount of unnecessary chemotherapy prescribed. In addition to the quality-of-life issues, the financial saving would be considerable. It is estimated that an outpatient course of six cycles of adriamycin/cyclophosphamide (AC) chemotherapy (a standard but by no means the most expensive schedule), costs at least £2400 to deliver. Assuming that around 50% of cases diagnosed receive adjuvant chemotherapy, this will be administered to around 20 000 women with breast cancer each year in the UK. Expression profiling is an expensive technology but if adjuvant chemotherapy use was reduced by 70% (see Figure 1), saving at the very least 33 million pounds, it would appear much more financially viable as a routine clinical test. If adjuvant use of taxanes increases, the saving would escalate considerably. It is quite possible that molecular profiling will identify a single (or handful) of markers that are sufficient to predict prognosis with great accuracy, which would represent a far simpler and cheaper test. Chemosensitivity testing would result in a further reduction in the amount of chemotherapy prescribed. Inevitably, there will be a considerable number of women who will be identified as being resistant to conventional cytotoxics; these patients should be considered for entry into trials of new agents. In addition to quality-of-life improvements, this patient group will have the opportunity of benefiting from exposure to potentially effective novel agents.

Molecular profiling may be useful in the future in improving the targeting of treatments in other areas of breast cancer treatment. With the widespread introduction of mammographic screening, the diagnosis of DCIS has increased markedly. It is difficult to predict those cases at risk of progression to invasive disease with the result that many women receive adjuvant radiotherapy unnecessarily. Molecular profiling may allow the high-risk subtypes to be identified more accurately. Similarly, accurate identification of the subtype of metastatic breast cancer patients who are at high risk of forming cerebral metastases would allow exploration of the use of prophylactic cerebral irradiation as a means of preventing their development.

Genomic or proteomic profiling will also make a contribution to the subclassification of breast cancer. Screening the genome for deletions and amplifications has been used successfully to identify abnormalities associated with survival (Aubele *et al*, 2002) that may have a role in predicting patients at risk of relapse. There is also considerable interest in the use of proteomic profiling of circulating blood as a screening tool (Petricoin *et al*, 2002) which, in theory, would allow cancer to be diagnosed by a blood test.

Although the molecular profile of the tumour is a major determinant of disease progression and response to treatment, other factors may be of considerable importance. For example, treatment response and toxicity may be influenced by drug metabolism and possibly the inherent activity of DNA repair pathways; these, in turn, are influenced by the germline genetic profile of the patient. Assessment of such parameters (SNPs) has the potential for predicting adverse drug reactions and for obtaining a more accurate prediction of treatment response in combination with the profile of the tumour.

The survival of breast cancer has improved markedly over the last three decades, but this has been associated with ‘over treatment’ for many patients. More effective and less toxic agents are urgently required to improve the overall survival rates and reduce side effects but in the meantime molecular profiling may play a role in reducing the delivery of nonbeneficial treatment.
